# Cholecystectomy in the Pediatric Population—What Has Changed in Recent Decades? Insight from a Tertiary Pediatric Referral Center

**DOI:** 10.3390/epidemiologia7020047

**Published:** 2026-04-02

**Authors:** Tal Weiss, Yael Dreznik, Dragan Kravarusic, Samah Hayek

**Affiliations:** 1 Department of Epidemiology and Preventive Medicine, School of Public Health, Gray Faculty of Medical and Health Sciences, Tel Aviv University, Tel Aviv 6139001, Israel; talmager@mail.tau.ac.il; 2 School of Medicine, Gray Faculty of Medical and Health Sciences, Tel Aviv University, Tel Aviv 6139001, Israel; yael.dreznik@gmail.com (Y.D.); dragank@clalit.org.il (D.K.); 3 Department of General Surgery, Hillel Yaffe Medical Center, The Rapaport School of Medicine, Technion, Haifa 3525433, Israel; 4 Department of Pediatric and Adolescent Surgery, Schneider Children’s Medical Center of Israel, Petah-Tiqwa 4920235, Israel; 5 Clalit Innovation, Clalit Health Services, Ramat-Gan 5252247, Israel

**Keywords:** pediatric cholecystectomy, cholelithiasis, hemolytic disorders, childhood obesity, biliary dyskinesia, gallbladder disease

## Abstract

Background: Symptomatic cholelithiasis is the leading indication for pediatric cholecystectomy. While historically linked to hemolytic disorders, non-hemolytic gallbladder disease in children has become increasingly common in recent decades. Objective: The objective of this study was to describe the distribution and temporal trends of indications for cholecystectomy among children (ages ≤ 19 years) undergoing surgery at a tertiary pediatric center in Israel and to compare clinical presentation between hemolysis-related and non-hemolysis-related cases. Methods: We conducted a retrospective observational cohort study of all pediatric patients who underwent cholecystectomy at Schneider Children’s Medical Center between 2011 and 2024. Patients with congenital biliary tract anomalies or biliary tract neoplasms were excluded. Results: A total of 199 cholecystectomies were performed (median age 13.4 years). Hemolysis-related cholelithiasis accounted for 34.2% of cases; five patients (2.5%) had gallbladder polyps or other benign lesions, while the remaining patients had non-hemolysis-related cholelithiasis. No cases of biliary dyskinesia were identified. The proportion of non-hemolysis-related cholecystectomies remained stable over time. Among symptomatic patients, the rate of choledocholithiasis was significantly higher in the hemolysis-related group compared to the non-hemolysis group (27% vs. 7.9%, *p* = 0.004). No statistically significant association was observed between obesity and increased disease severity or adverse outcomes. Conclusion: Unlike trends reported in some Western countries, the number of cholecystectomies performed for non-hemolysis-related cholelithiasis in our single-center cohort did not increase over time. Hemolysis-related disease remains a leading indication for pediatric cholecystectomy. Prophylactic surgery may help prevent biliary complications in this group while symptomatic patients have substantial complication rates.

## 1. Introduction

Historically, pediatric gallbladder disease has been predominantly attributed to cholelithiasis (gallstone formation) associated with hemolytic disorders, such as hereditary spherocytosis [1]. Chronic hemolysis increases bilirubin production, leading to bile supersaturation and the formation of black pigment gallstones [2]. These pigment stones are typically composed of calcium bilirubinate and other calcium salts and are often small and irregular in shape, which facilitates their migration into the bile duct and may increase the risk of complications such as pancreatitis or cholangitis [3].

In recent decades, however, the incidence of gallbladder disease unrelated to hemolytic conditions has increased, paralleling increased rates of pediatric cholecystectomy [4,5]. This shift has been partially attributed to the growing prevalence of pediatric obesity, which is associated with a higher risk of cholesterol biliary stones [6,7,8,9], thereby altering the etiology profile of pediatric gallbladder disease to more closely resemble that seen in adults [10].

Additionally, the increased diagnosis of biliary dyskinesia, a functional disorder of the gallbladder characterized by impaired motility, leading to inadequate bile ejection [11], has contributed to rising cholecystectomy rates, particularly in the United States [5,12]. However, in other Western countries, biliary dyskinesia remains a rare indication for surgery, and its status as a distinct clinical entity remains debated [13].

Symptomatic cholelithiasis presenting as biliary colic, cholecystitis, choledocholithiasis, cholangitis, or pancreatitis is the primary indication for cholecystectomy in children regardless of the underlying etiology. Asymptomatic cases are generally not considered an indication for cholecystectomy, except in children with hemolytic disorders, where it may be considered a relative indication with or without concomitant splenectomy [14].

Multiple risk factors for cholelithiasis have been identified, including cystic fibrosis, bariatric surgery, certain medications (e.g., hormonal contraceptives and ceftriaxone), and total parenteral nutrition (TPN) [7]. Congenital biliary tract malformations may also predispose patients to gallstone formation by promoting bile stasis and impaired bile flow [15,16]. Obesity is considered a major risk factor for cholelithiasis as it promotes cholesterol supersaturation of bile [17].

Childhood and adolescent obesity have emerged as a global epidemic [18], yet few studies have examined its role in the increasing incidence of gallbladder disease in children [6,7,8,9]. In Israel, like other Western countries, childhood obesity rates have continued to increase, particularly among lower socioeconomic groups [19].

In the current study, we aim to describe the distribution of indications for cholecystectomy among children undergoing surgery in a tertiary center over the past 14 years. Furthermore, the study aims to evaluate differences in the manifestation of cholelithiasis between patients with hemolysis-related cholelithiasis and non-hemolysis cholelithiasis.

### Impact Statement

In contrast to trends reported in Western countries, hemolysis remains a leading cause of pediatric cholecystectomy in Israel, with no increase in obesity-related disease or biliary dyskinesia. These findings highlight regional differences in pediatric gallbladder disease and emphasize the need for context-specific clinical guidelines and further research.

## 2. Methods

### 2.1. Study Design and Population

We conducted a retrospective observational cohort study evaluating the indications and clinical manifestations of biliary disease among pediatric patients undergoing cholecystectomy at Schneider Children’s Medical Center (SCMC), a tertiary pediatric referral hospital in Israel, between 2011 and 2024. SCMC is the largest pediatric hospital in Israel and provides clinical care to children from across the country. All pediatric patients who underwent cholecystectomy for any indication during the study period were eligible for inclusion. Patients were excluded if they had congenital anomalies of the biliary tract or biliary tract neoplasms.

The primary objective of the study was to describe the distribution and temporal trends of indications for cholecystectomy among children undergoing surgery at our institution over the 14-year study period. The secondary objectives were to compare the clinical manifestations of biliary disease (biliary colic, acute cholecystitis, pancreatitis, choledocholithiasis, and asymptomatic cholelithiasis) between patients with hemolysis-related and non-hemolysis-related cholelithiasis, and to evaluate the association between obesity and perioperative outcomes.

Based on prior studies [20,21], a sample of 140 patients was estimated to provide sufficient statistical power (80%) to detect significant trends using a Cochran–Armitage test (alpha = 0.05). Our final cohort included 199 patients who met the eligibility criteria, ensuring adequate statistical power.

### 2.2. Ethics Approval and Consent

This retrospective study was conducted in accordance with relevant guidelines and regulations. The study protocol was approved by the Institutional Helsinki Committee at Schneider Children’s Medical Center (number RMC-0367-24 and date of approval 16 July 2024). Given the retrospective design and exclusive use of de-identified electronic medical records, the requirement for informed consent was waived by the same ethics committee.

### 2.3. Clinical Characteristics and Outcomes

Demographic and clinical data were extracted from the SCMC electronic health records (EHR). Outcome variables included (1) type of biliary disease (i.e., hemolysis-related cholelithiasis, non-hemolysis-related cholelithiasis, biliary dyskinesia, and other benign causes) and (2) clinical manifestations of biliary disease (i.e., biliary colic, acute cholecystitis, choledocholithiasis, gallstone pancreatitis, and cholangitis).

Obesity was defined based on either a documented diagnosis in the medical record or body mass index (BMI). A BMI above the 95th percentile for age and sex was classified as obesity, while a BMI between the 85th and 95th percentiles was classified as overweight. BMI percentiles were determined using the CDC BMI-for-Age Percentile Charts [22].

Additional variables included age, sex, ethnicity, risk factors (oral contraceptive use, total parenteral nutrition [TPN], and ceftriaxone exposure), comorbidities (irritable bowel disease [IBD], prematurity, and neoplasms), previous abdominal surgeries, surgical indications, operative details, and postoperative complications. Because multiple risk factors may coexist in individual patients, these variables were recorded descriptively and were not used to create mutually exclusive etiological classifications. Complicated biliary disease was defined as the presence of choledocholithiasis, cholangitis, or pancreatitis.

### 2.4. Statistical Analysis

Descriptive statistics were used to summarize the study population. Categorical variables are presented as frequencies and percentages, and the chi-square test or Fisher’s exact test was conducted for comparison between groups. Continuous variables were assessed for normality. Normally distributed variables are presented as means with standard deviations and were compared using the independent t-test. Non-normally distributed variables are presented as medians with interquartile ranges (IQR, 25th–75th percentiles) and were compared using the Wilcoxon rank-sum test. Temporal trends in disease etiology were assessed using the Cochran–Armitage test for trend.

Associations between categorical predictors (e.g., hemolysis status and obesity) and binary outcomes (e.g., complicated disease or postoperative complications) were evaluated using chi-square tests. Multivariable logistic regression models were used to assess the association between obesity and clinical outcomes, including complication rates, operative time, hospital length of stay, and presence of complicated biliary disease. All models were adjusted for age, sex, and ethnicity to control for potential confounders.

Analyses were performed using R software (version 4.3.1), with a *p*-value < 0.05 considered statistically significant.

## 3. Results

A total of 199 pediatric patients underwent cholecystectomy at SCMC between 2011 and 2024. The median duration of follow-up was 5.9 years (interquartile range [IQR], 2.7–8.5 years). The median age at surgery was 13.4 years (IQR 9.0–16.2), and 68.3% (n = 136) were female (Table 1). Among the study participants, 68.8% were of normal weight, 4.0% had overweight, and 27.1% had obesity.

The total number of cholecystectomies increased over the study period, with a temporary decline observed in the number of procedures between 2019 and 2021. Supplemental Figure S1 shows the annual number of cholecystectomies performed during the study period, along with the distribution of hemolysis-related and non-hemolysis-related cases.

Hemolysis-related cholelithiasis was identified in 34.2% of patients, while 63.3% had non-hemolysis-related cholelithiasis. Hemolytic disorders included hereditary spherocytosis (n = 49), thalassemia (n = 9), sickle cell disease (n = 6), sickle cell thalassemia (n = 1), autoimmune hemolytic anemia (n = 1), G6PD deficiency (n = 1), and stomatocytosis (n = 1).

Additional stone-related risk factors were uncommon, including oral contraceptive use (1%), ceftriaxone exposure (1%), total parenteral nutrition (2%), prior bariatric surgery (4%), and other forms of rapid weight loss (1.5%). A small proportion of patients (2.5%) underwent cholecystectomy for non-cholelithiasis indications, including gallbladder polyps and other benign lesions. No patient underwent cholecystectomy for biliary dyskinesia.

Most procedures were performed via laparoscopic cholecystectomy (89.9%). Combined procedures included laparoscopic cholecystectomy with splenectomy in 8.5% of cases and with sleeve gastrectomy in 1%. One patient underwent an open cholecystectomy combined with gastric bezoar extraction. The mean operative time was 137 min (SD ± 76), and postoperative complications occurred in 4.0% of patients. These included six patients with Clavien-Dindo grade II complications and two patients with grade IIIb complications.

The study included 26.6% Arab and 73.4% Jewish patients. No significant differences were observed between Jewish and Arab patients across demographic and clinical factors (Supplemental Table S1). No significant temporal trends were observed over time in age, gender, or ethnicity. However, the prevalence of obesity increased from 9.1% in the 2011–2012 period to 41% in the 2021–2022 period (*p* for trend = 0.008), while the proportion of hemolysis-related cholelithiasis remained stable (Supplemental Table S2).

Among patients with hemolytic disorders, 32 (47%) underwent prophylactic cholecystectomy while asymptomatic; of these, 21 underwent isolated cholecystectomy and 11 underwent combined cholecystectomy and splenectomy. The rate of biliary colic was significantly higher in the non-hemolytic group (63% vs. 29%, *p* < 0.001). No significant difference was observed in the rate of complicated disease between the two groups (20% vs. 22%, *p* = 0.820) (Table 2).

In symptomatic patients, biliary colic remained the most common indication in both groups. Although not statistically significant, among symptomatic patients only, a higher proportion of patients with hemolysis-related cholelithiasis presented with complicated disease (37.8% vs. 22.2%, *p* = 0.089) (Supplemental Table S3). Choledocholithiasis was significantly more common among symptomatic patients with hemolysis-related cholelithiasis (27.0% vs. 7.9%, *p* = 0.004). No significant differences were observed for biliary pancreatitis or cholangitis. None of the patients who underwent prophylactic cholecystectomy while asymptomatic were subsequently readmitted with biliary complications such as choledocholithiasis or pancreatitis during the follow-up period.

When stratified by obesity status, no statistically significant differences were observed in complication rate (3.7% vs. 4.1%), operative time (140 ± 66 vs. 136 ± 80 min), or hospital length of stay (2.76 ± 1.96 vs. 2.88 ± 2.31 days). When the analysis was restricted to patients who underwent cholecystectomy alone (excluding combined procedures), the results remained similar, with no statistically significant differences in complication rate (3.7% vs. 3.1%), operative time (130 ± 62 vs. 127 ± 64 min), or hospital length of stay (2.66 ± 2.18 vs. 2.72 ± 2.50 days). The rate of complicated biliary diseases was similar between groups (22% in patients with obesity vs. 21% in patients without obesity) (Table 3). After adjusting for age, sex, and ethnicity, obesity was not significantly associated with complication rate, operative time, length of stay, or presence of complicated biliary diseases (Table 4).

## 4. Discussion

Pediatric cholecystectomy rates have increased worldwide, largely driven by non-hemolysis-related etiologies such as obesity and biliary dyskinesia. In contrast, our study, conducted at Israel’s largest pediatric referral hospital over a 14-year period, found that hemolysis-related disease remains a predominant indication, with no significant rise in non-hemolytic cholelithiasis. These findings provide a unique perspective on the patterns of biliary disease among children undergoing cholecystectomy, particularly within the context of a public healthcare system.

The annual number of cholecystectomies generally increased during the study period, with a temporary decline between 2019 and 2021, which may reflect the impact of the COVID-19 pandemic on elective surgical activity. As SCMC serves as a major national referral center, the observed patterns may reflect broader clinical trends; however, national-level data for Israel are currently unavailable, limiting population-level inference. Similar increases have been reported internationally [20,23,24].

In our cohort, obesity prevalence was 27%, with combined rates of overweight and obesity reaching 31%. These rates are notably higher than those reported in the general Israeli pediatric population, where obesity prevalence is up to 7.7% among 7-year-old children [25] and up to 21.4% among 17-year-old males [26]. This discrepancy likely reflects the well-established association between obesity and cholelithiasis, which has been documented in both adults and children [7,27].

Contrary to international reports, we did not observe a significant increase over time in the proportion of non-hemolysis-related cholelithiasis. Studies from North America have linked the increasing cholecystectomy rates to increasing obesity, oral contraceptive use, and dietary changes [4,9,25]; this trend was not observed in our study. Although obesity rates increased among children undergoing cholecystectomy, this was not accompanied by a corresponding rise in the proportion of cases attributed to obesity-related cholelithiasis. Differences in dietary patterns, lifestyle factors and healthcare practices may contribute to this discrepancy.

Another possible explanation may relate to differences in surgical indications across countries. Internationally, the diagnosis of biliary dyskinesia and the use of cholecystectomy for its surgical management have increased [12,13,26]. In the United States, biliary dyskinesia accounts for up to 30–50% of pediatric cholecystectomy indications [12,13,26,28]. Nevertheless, in our cohort from the largest tertiary pediatric hospital in Israel, no patient underwent cholecystectomy for biliary dyskinesia during the 14-year study period, highlighting how uncommon this indication is in Israel.

Interestingly, limited data exist on the frequency of biliary dyskinesia outside the United States. A comparative analysis using national surgical registries from Sweden, Norway, and Poland found that the annual rate of cholecystectomy for biliary dyskinesia was less than 25 per 1,000,000 people in these European countries compared to over 85 per 1,000,000 in the United States [13]. Furthermore, despite its rising prevalence in the United States, the current literature on biliary dyskinesia in the pediatric population remains limited, with all available studies being retrospective [29]. Since the diagnosis of biliary dyskinesia in children remains controversial and lacks universally accepted diagnostic criteria, biliary dyskinesia is rarely pursued as a diagnostic entity in our clinical practice, and cholecystectomy was not performed for this indication in our cohort.

Differences in healthcare infrastructure may contribute to the discrepancy between the marked increase in cholecystectomy for non-hemolytic gallbladder disease reported in the international literature, and the relatively stable pattern observed in our Israeli cohort. One possible explanation is the structure of the Israeli healthcare system, which provides universal coverage with minimal private sector influence, potentially reducing the frequency of early elective cholecystectomy in the absence of overt pathology. While the growing availability and use of abdominal ultrasound have led to increased detection of asymptomatic cholelithiasis [30], private healthcare systems may be more inclined toward early surgical intervention. In contrast, a public healthcare model may favor a more conservative, symptom-driven approach. Nevertheless, this interpretation remains speculative and cannot be confirmed by the data presented in this study. Other factors may also contribute to these differences, including variation in diagnostic criteria, differences in diagnostic resources, and institutional clinical practice patterns.

Arab children comprised 26% of our cohort, higher than their proportion in the Israeli general population [31]. This may reflect a higher prevalence of obesity [32] or possibly genetic hemolytic disorders in this group; however, we did not find data supporting this hypothesis. Nevertheless, no significant difference in biliary disease etiology was observed between Arab and Jewish patients.

Distinct patterns of biliary disease manifestation were observed between patients with and without hemolytic disorders. Less than half of hemolysis-related cases underwent prophylactic cholecystectomy while asymptomatic despite some conflicting guideline recommendations [33,34,35,36]. This may reflect a tendency to delay cholecystectomy until splenectomy or until symptom onset. The overall rate of complicated disease was similar between the hemolytic and non-hemolytic groups; however, among symptomatic patients, choledocholithiasis was significantly more frequent in those with hemolysis-related cholelithiasis. This may reflect the inherent tendency of pigment stones associated with hemolytic disorders to migrate into the common bile duct. Alternatively, it may partly result from lower diagnostic thresholds in these patients, whose chronically elevated bilirubin levels often prompt earlier imaging and therefore greater detection of bile duct stones. Notably, no biliary complications were observed among patients who underwent prophylactic cholecystectomy during the available follow-up period, supporting previous evidence suggesting that prophylactic cholecystectomy may help prevent biliary complications in patients with hemolysis-related cholelithiasis.

Contrary to previous reports linking obesity with more severe biliary disease [7,33,34], our study did not observe higher rates of choledocholithiasis, cholangitis, or pancreatitis among patients with obesity. Similarly, no statistically significant differences were found in operative time, hospital stay, or complication rates between patients with and without obesity. These findings remained consistent even when restricting the analysis to patients undergoing cholecystectomy alone, excluding combined procedures such as splenectomy or sleeve gastrectomy. These results contrast with the findings reported in adult populations [37] and may be attributable to the younger age and lower comorbidity burden of pediatric patients. Nevertheless, the relatively small number of patients with obesity in our cohort may limit the statistical power to detect modest associations, and these findings should therefore be interpreted with caution.

This study has several limitations. As a single-center analysis conducted at a national tertiary pediatric referral hospital, referral bias is possible, with potential overrepresentation of patients with complex disease or underlying hemolytic disorders. Information bias may also exist due to the retrospective design and reliance on electronic medical records, which may contain inconsistent documentation of symptoms, BMI, and complications. Although follow-up information was available for most patients through manual review of institutional records and the national electronic medical record system (“Ofek”), complete long-term follow-up cannot be guaranteed for all patients. Additionally, because national population-based data on pediatric cholecystectomy are not available in Israel, our analysis reflects institutional trends at a tertiary referral center rather than changes in population-level incidence. Important clinical factors such as gallstone composition, socioeconomic background, and genetic predisposition were not assessed. Furthermore, changes in diagnostic practices or surgical indications at our institution over the 14-year study period may have influenced patient selection and management. Consequently, the generalizability of these findings to other institutions or healthcare systems may be limited. Finally, because the study did not include children with biliary disease who were managed non-operatively, we could not compare the characteristics of operated versus non-operated patients; therefore, demographic and etiologic distributions in this cohort may partly reflect referral and surgical selection patterns at a tertiary center.

## 5. Conclusions

In conclusion, despite international trends of increasing cholecystectomy for non-hemolytic and obesity-related gallbladder disease in children, hemolysis-related cholelithiasis remains a predominant indication for cholecystectomy in our cohort. Prophylactic surgery may help prevent biliary complications in this group, while symptomatic patients have substantial complication rates, particularly choledocholithiasis.

Although obesity is increasingly prevalent among pediatric cholecystectomy patients, we did not observe a statistically significant association with worse clinical or surgical outcomes. Because this study included only children undergoing cholecystectomy, the findings describe the surgical case mix rather than the full spectrum of pediatric biliary disease.

Future multicenter prospective studies are needed to confirm these findings and guide optimal management strategies for children with cholelithiasis.

## Figures and Tables

**Table 1:** Demographics and clinical characteristics of patients who underwent cholecystectomy at Schneider medical center between 2011 and 2024.

Age	13.4 (9–16.2)
Female gender	136 (68.3%)
Arabic ethnicity	53 (26.6%)
Weight class	
Normal weight	137 (68.8%)
Overweight	8 (4%)
Obese	54 (27.1%)
Disease etiology	
Hemolysis-related cholelithiasis	68 (34.2%)
Non-hemolysis-related cholelithiasis	126 (63.3%)
Other	5 (2.5%)
Other stone risk factors	
OCP use	2 (1%)
Ceftriaxone	2 (1%)
TPN	4 (2%)
Previous bariatric surgery	8 (4%)
Other weight loss	3 (1.5%)
Operative method	
Laparoscopic cholecystectomy	179 (89.9%)
Open cholecystectomy	1 (0.5%)
Laparoscopic cholecystectomy and splenectomy	17 (8.5%)
Laparoscopic cholecystectomy and sleeve gastrectomy	2 (1%)
Operation time in minutes (±SD)	137 (±76)
Post operation complications	8 (4%)

**Table 2:** Indication for surgery in children with hemolysis-related cholelithiasis and non-hemolysis-related cholelithiasis.

	Hemolysis-Related Cholelithiasis n = 68	Non-Hemolysis-Related Cholelithiasis n = 126	
Asymptomatic cholelithiasis	32 (47%)	0 (0%)	<0.001
Without splenectomy	21 (30%)	0 (0%)	
With splenectomy	11(16%)	0 (0%)	
Biliary colic	20 (29%)	80 (63%)	<0.001
Acute cholecystitis	2 (2.9%)	13 (10%)	0.091
Complicated disease	14 (20%)	28(22%)	0.820
Choledocholithiasis	10 (14%)	10 (7.9%)	0.146
Biliary pancreatitis	4 (5.9%)	16 (13%)	0.215
Cholangitis	0 (0%)	2 (1.6%)	0.542
Other	0 (0%)	5 (4%)	0.164

Note. Percentages are calculated within each etiologic group (column percentage).

**Table 3:** Comparison between pediatric patients with and without obesity who underwent laparoscopic cholecystectomy.

	Patients without Obesity n = 145	Patients with Obesity n = 54	*p*-Value
Complicated biliary disease	30 (21%)	12 (22%)	>0.9
Operation time mean (SD)	136 ± 80	140 ± 66	0.756
Length of stay mean (SD)	2.88 ± 2.31	2.76 ± 1.96	0.708
Complication rate mean (SD)	6 (4.1%)	2 (3.7%)	>0.9

Note. Percentages are calculated within each weight group (column percentage).

**Table 4:** Association between obesity and clinical outcomes.

	Adjusted Effect Size (95% CI)	*p*-Value
Complicated biliary disease	OR = 1.21 (0.52–2.72)	0.665
Operation time	Beta = 7.26 (−19.14–33.67)	0.588
Length of stay	Beta = 0.15 (−0.57–0.88)	0.676
Complication rate	OR = 0.25 (0.01–1.51)	0.203

Note. **OR** = odds ratio estimated from the multivariable logistic regression model; **Beta** = regression coefficient estimated from the multivariable linear regression model; **CI** = confidence interval.

## Data Availability

The data presented in this study are available on request from the corresponding author due to patient privacy regulations and institutional restrictions.

## Supplementary Materials

The following supporting information can be downloaded at https://www.mdpi.com/article/10.3390/epidemiologia7020047/s1, Figure S1: Number of cholecystectomies performed each year; Table S1: Demographics, clinical characteristics, and biliary disease etiology among patients of Jewish and Arab ethnicity; Table S2: Demographics, clinical characteristics, and biliary disease etiology across time periods; Table S3: Indication for surgery in children with symptomatic hemolysis-related cholelithiasis and non-hemolysis-related cholelithiasis.
