# Supplementary material for: Cholecystectomy in the Pediatric Population—What Has Changed in Recent Decades? Insight from a Tertiary Pediatric Referral Center
Source: Epidemiologia (Basel). 2026 Apr 2;7(2):47. doi: 10.3390/epidemiologia7020047 (PMC13114763; doi:10.3390/epidemiologia7020047)
Supplement: Supplementary file 1 [file epidemiologia-07-00047-s001.zip › epidemiologia-4175325-supplementary.pdf]

| **Supplemental table S1: Demographics, clinical characteristics, and biliary disease etiology among patients of Jewish and Arab ethnicity.** | | | |
|---|---|---|---|
| | Jewish Ethnicity (n=146) | Arabic Ethnicity (n=53) | *P*- value |
| Age | 13.6 (7.3, 16.1) | 13.1 (9.1, 16.4) | >0.9 |
| Male Gender | 50 (34%) | 13 (25%) | 0.3 |
| Overweight or Obese | 44 (31%) | 16 (33%) | >0.9 |
| Disease etiology | | | >0.9 |
| Hemolytic related cholelithiasis | 50 (34%) | 18 (34%) | |
| Non hemolytic related cholelithiasis | 92 (63%) | 34 (64%) | |
| Others | 4 (2.7%) | 1 (1.9%) | |

**Supplemental Table S2: Demographics, clinical characteristics, and biliary disease etiology across time periods.**

| | **2011-2012** N = 22 | **2013-2014** N = 17 | **2015-2016** N = 18 | **2017-2018** N = 44 | **2019-2020** N = 27 | **2021-2022** N = 34 | **2023-2024** N = 37 | **P value*** |
|---|---|---|---|---|---|---|---|---|
| Age | 12.0 (7.1, 13.9) | 14.1 (10.5, 15.4) | 12.1 (9.1, 16.8) | 12.5 (7.4, 16.7) | 15.1 (10.1, 16.4) | 14.1 (10.9, 16.4) | 14.0 (8.4, 15.7) | P= 0.403 |
| Male gender (%) | 10 (45%) | 3 (18%) | 4 (22%) | 16 (36%) | 8 (30%) | 11 (32%) | 11 (30%) | P= 0.701 |
| Arabic Ethnicity | 3 (14%) | 3 (18%) | 4 (22%) | 14 (32%) | 7 (26%) | 10 (29%) | 12 (32%) | P= 0.087 |
| Overweight or Obese | 2 (9.1%) | 3 (18%) | 5 (28%) | 12 (27%) | 12 (44.7%) | 15 (43.9%) | 13 (35%) | P= 0.008 |
| Disease etiology | | | | | | | | P= 0.812 |
| Hemolytic related cholelithiasis | 8 (36%) | 5 (29%) | 9 (50%) | 15 (34%) | 7 (26%) | 9 (26%) | 15 (41%) | |
| Non hemolytic related cholelithiasis | 12 (55%) | 12 (71%) | 8 (44%) | 29 (66%) | 18 (67%) | 25 (74%) | 22 (59%) | |
| Others | 2 (9.1%) | 0 (0%) | 1 (5.6%) | 0 (0%) | 2 (7.4%) | 0 (0%) | 0 (0%) | |

*All p-values represent tests for linear trend across time periods using the Cochran-Armitage trend test, except for age which was analyzed using one-way ANOVA.

| **Supplemental table S3: Indication for surgery in children with symptomatic hemolysis-related cholelithiasis and non-hemolysis-related cholelithiasis.** | | | |
|---|---|---|---|
| | Hemolysis-related cholelithiasis N = 37 | Non-hemolysis-related cholelithiasis N = 126 | P-value |
| Biliary colic | 20 (54.1%) | 80 (63%) | 0.3 |
| Acute cholecystitis | 2 (5.4%) | 13 (10.3%) | 0.5 |
| Complicated disease | 14 (37.8%) | 28 (22.2%) | 0.089 |
| Choledocholithiasis | 10 (27.0%) | 10 (7.9%) | 0.004 |
| Biliary pancreatitis | 4 (10.8%) | 16 (12.7%) | >0.9 |
| Cholangitis | 0 | 2 (1.6%) | >0.9 |
| Other | 0 | 5 (4.0%) | >0.9 |

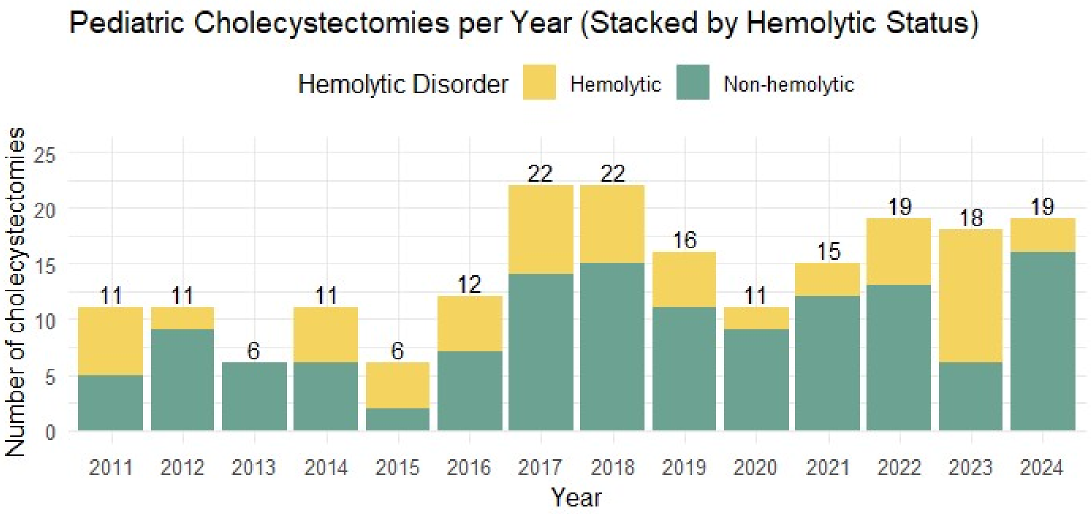


**Figure S1. Number of cholecystectomies performed each year.**
